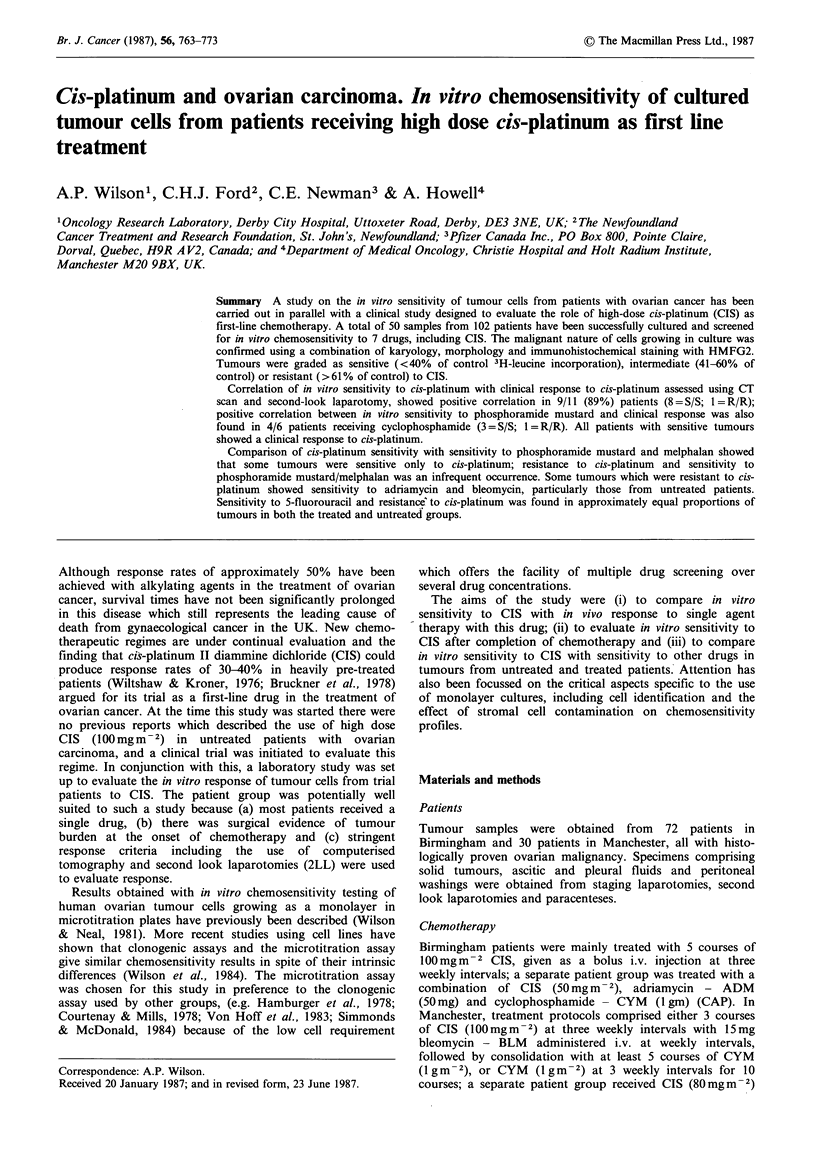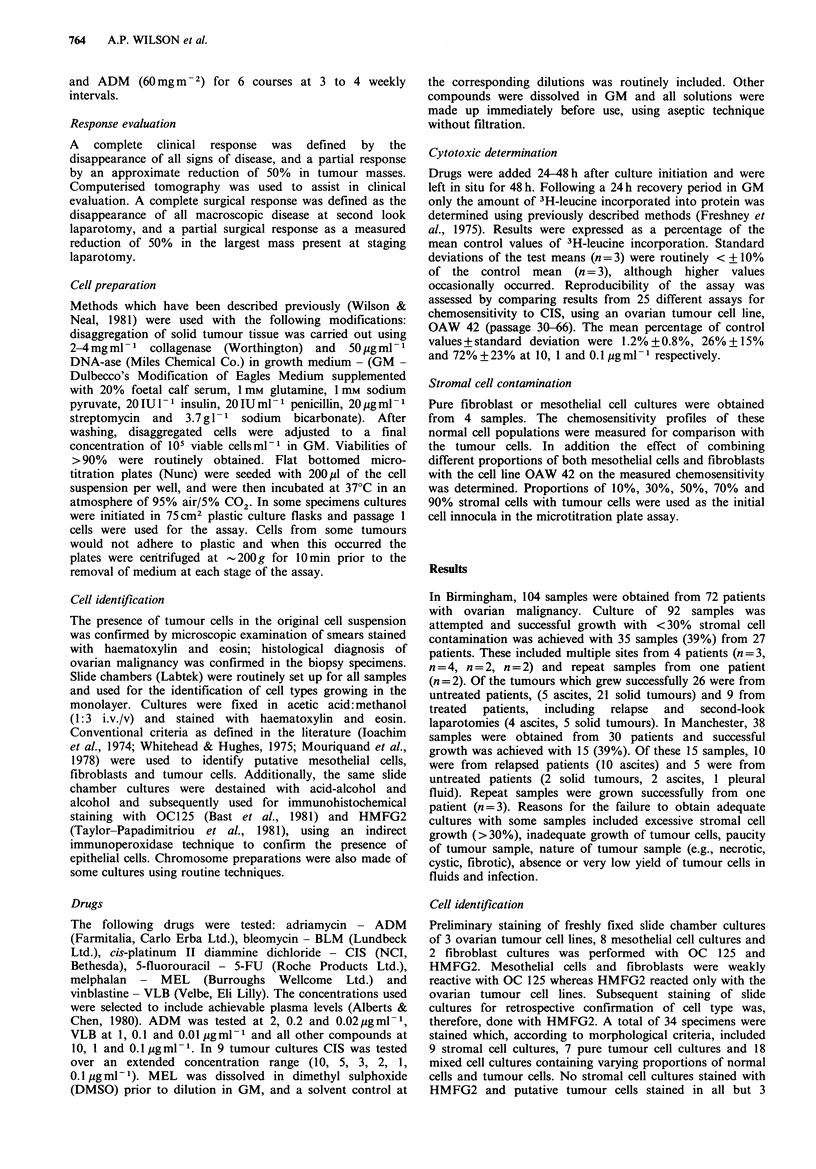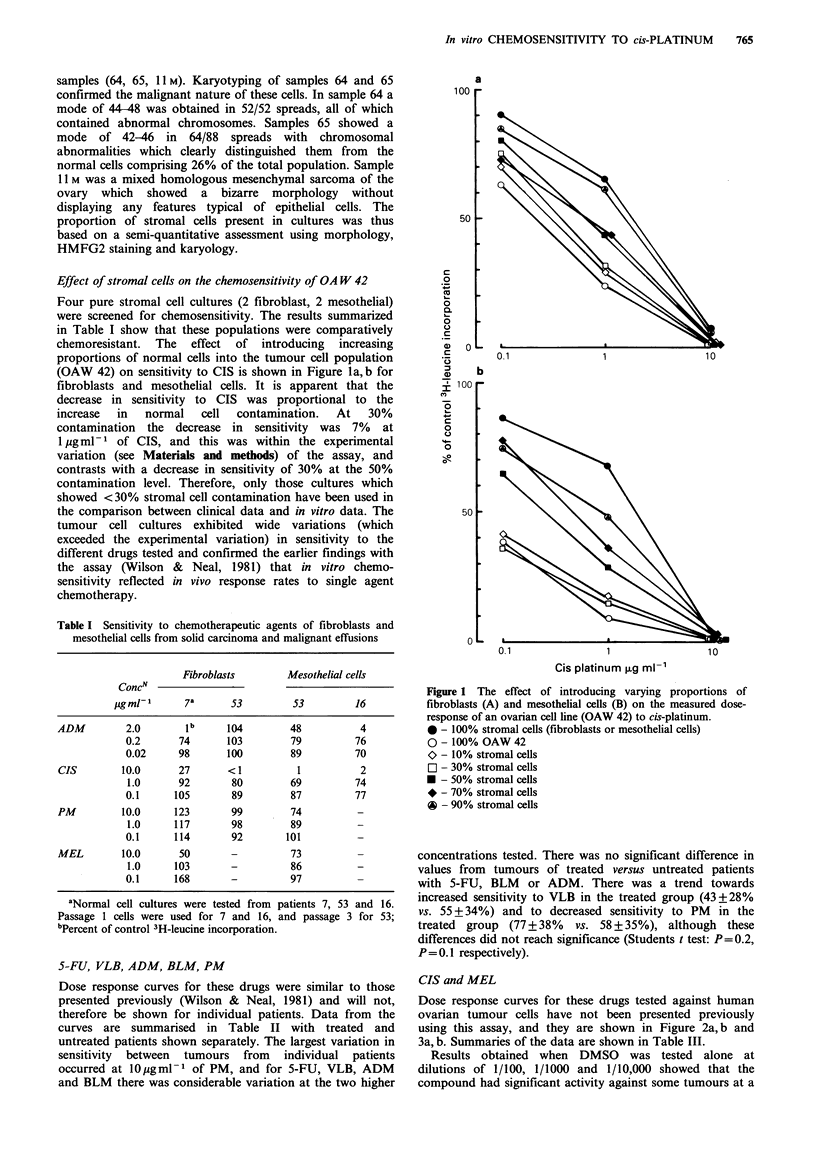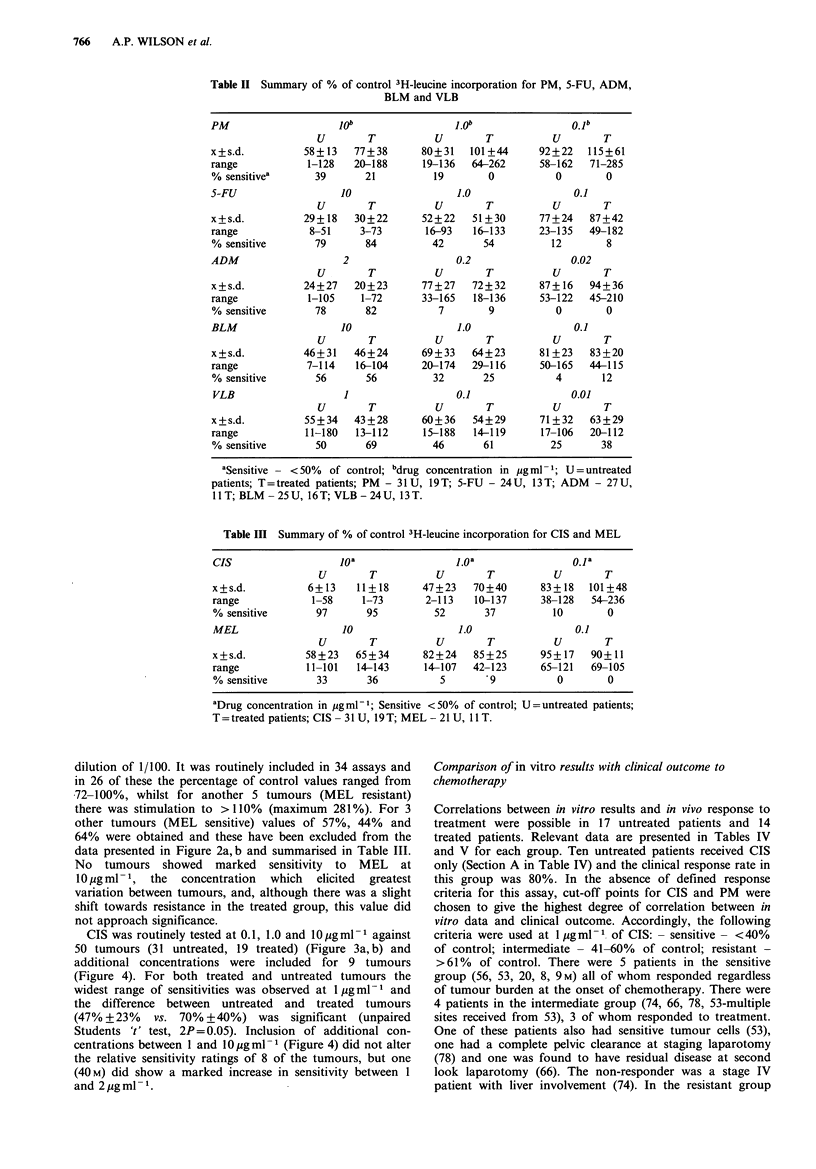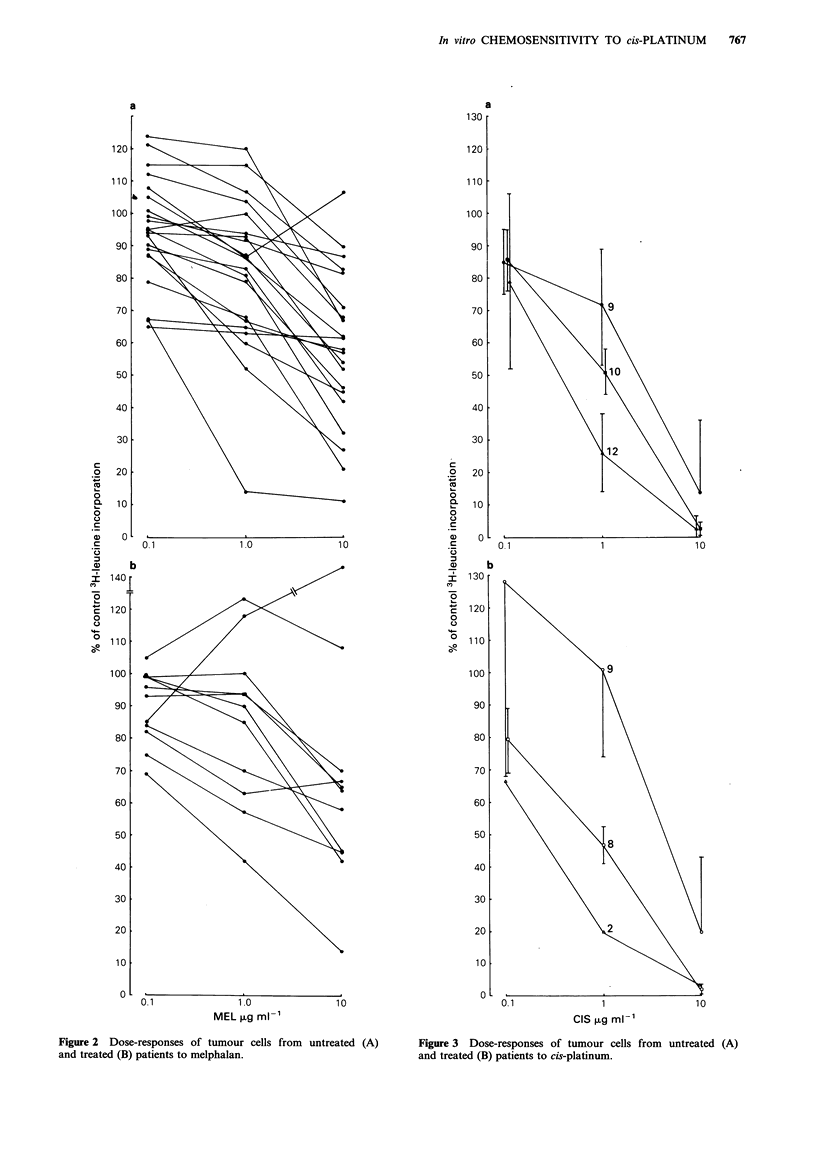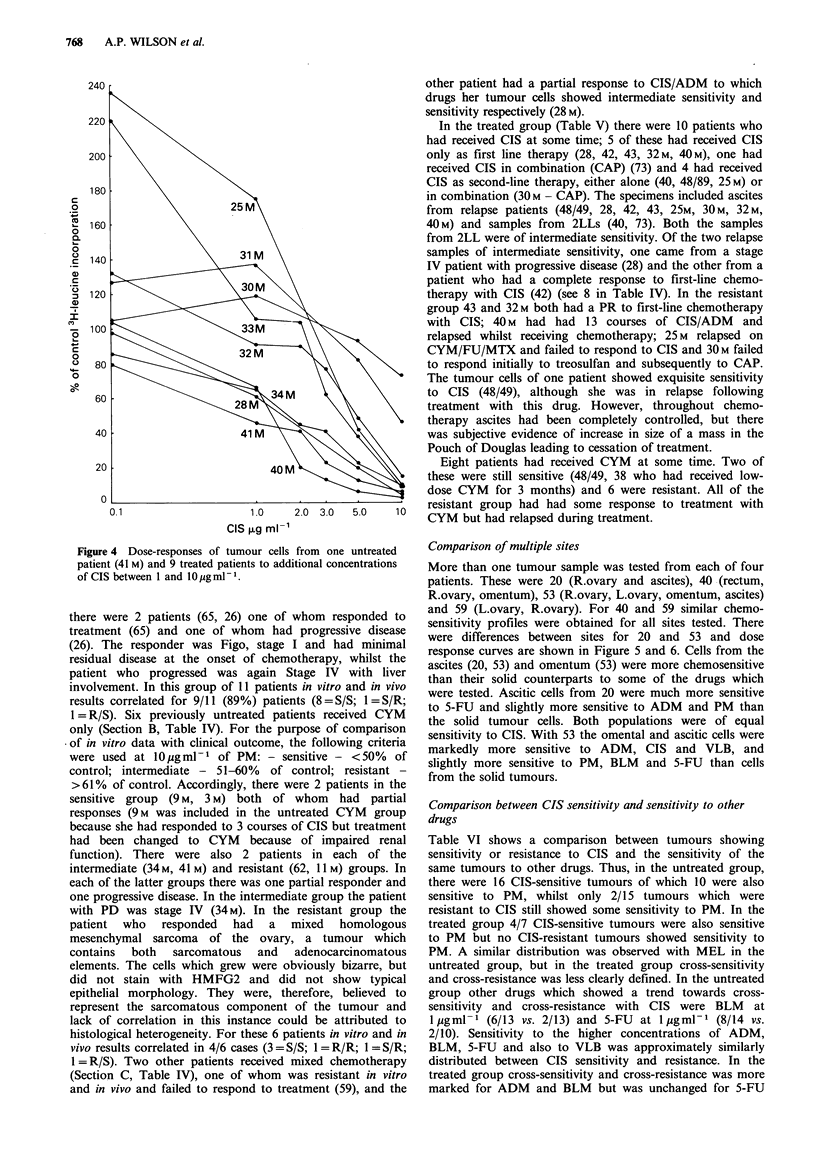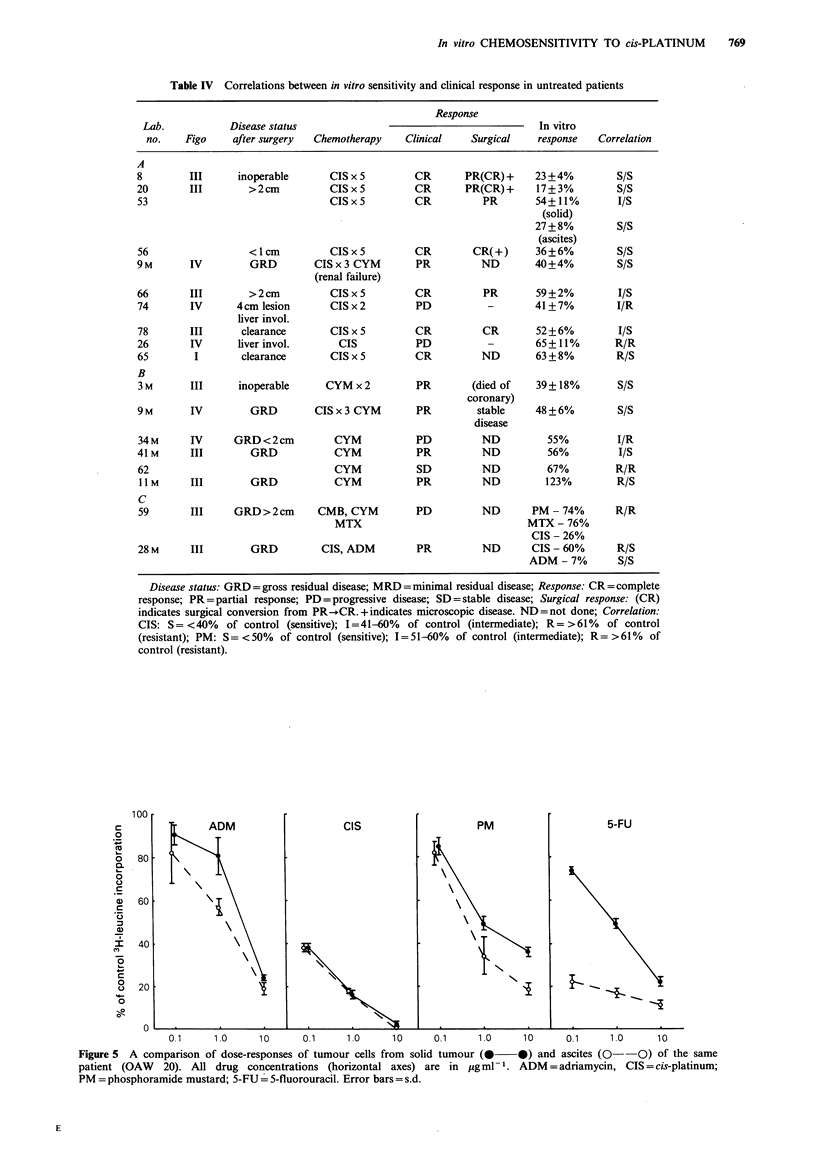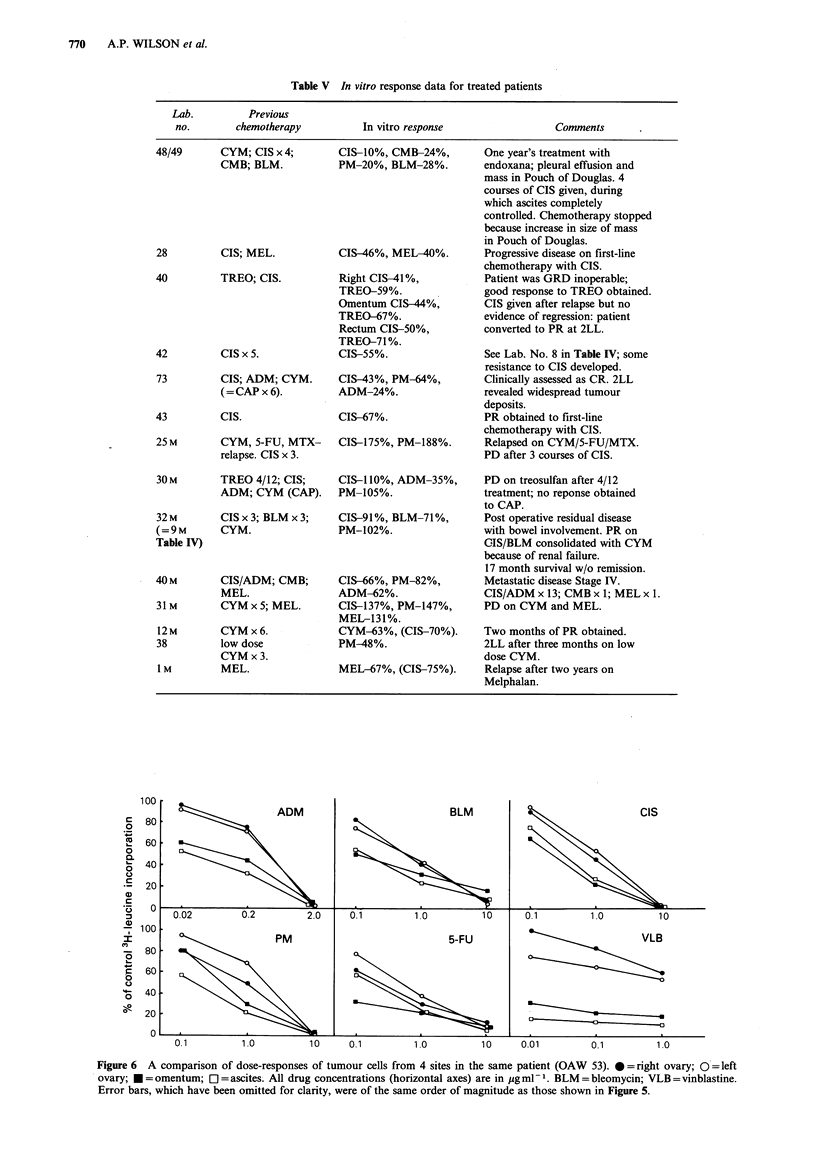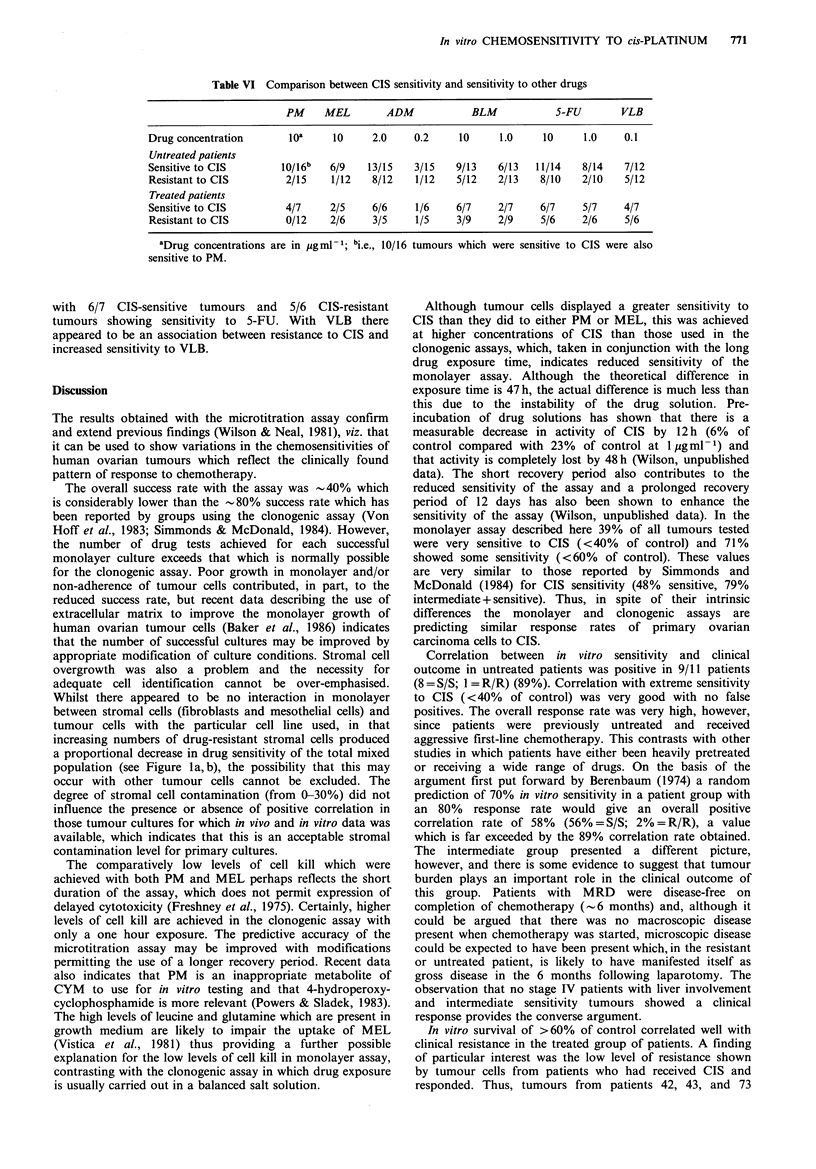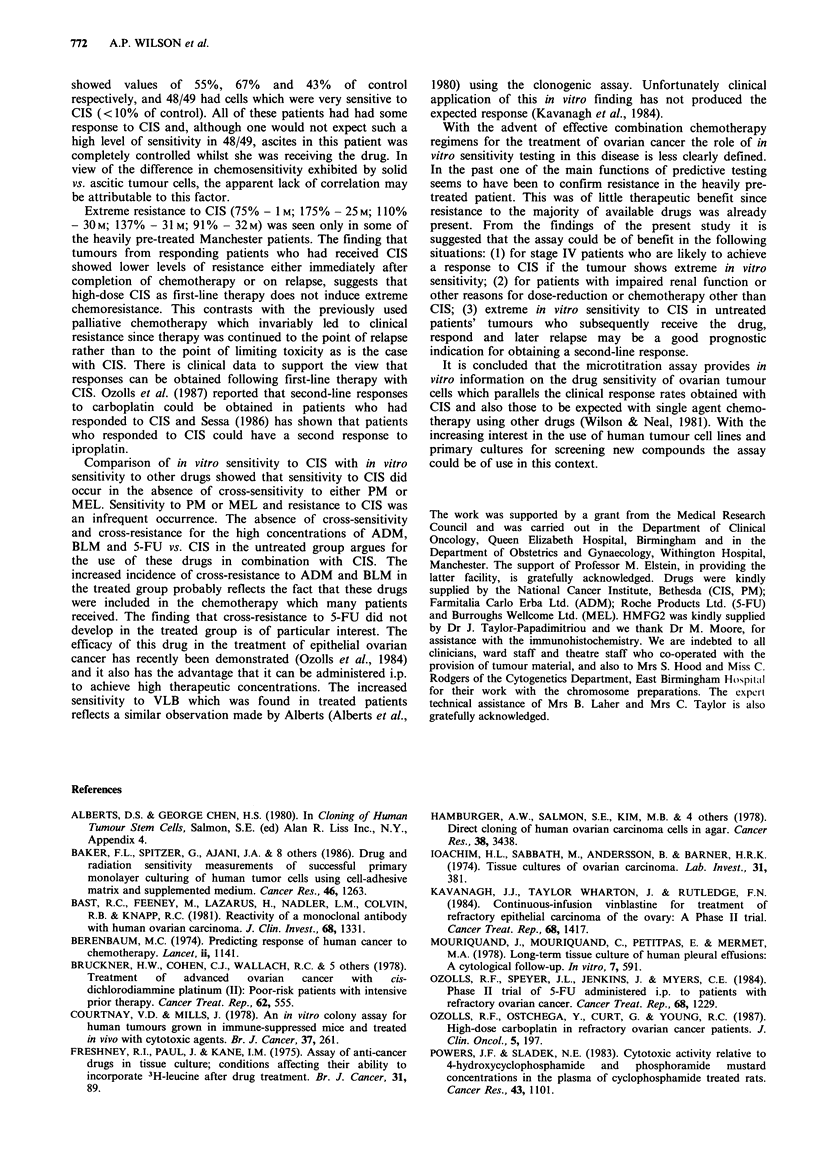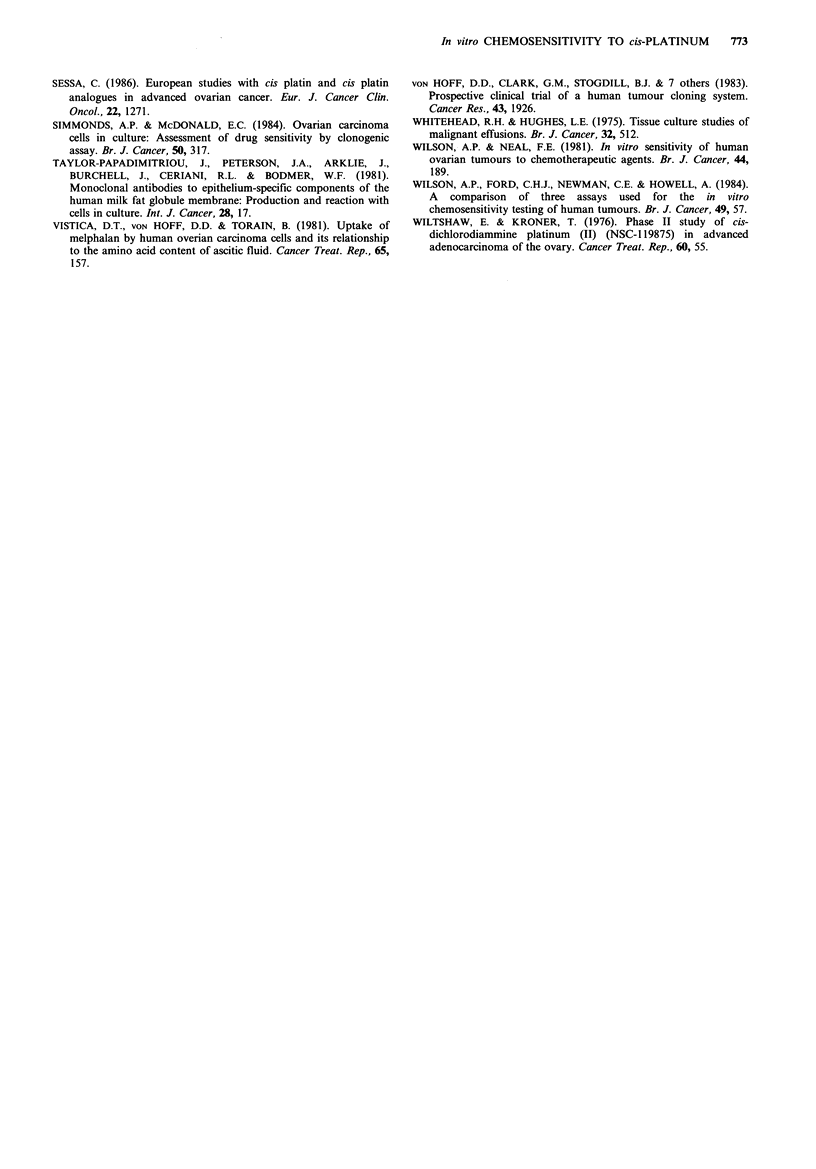# cis-platinum and ovarian carcinoma. In vitro chemosensitivity of cultured tumour cells from patients receiving high dose cis-platinum as first line treatment.

**DOI:** 10.1038/bjc.1987.285

**Published:** 1987-12

**Authors:** A. P. Wilson, C. H. Ford, C. E. Newman, A. Howell

**Affiliations:** Oncology Research Laboratory, Derby City Hospital, UK.

## Abstract

A study on the in vitro sensitivity of tumour cells from patients with ovarian cancer has been carried out in parallel with a clinical study designed to evaluate the role of high-dose cis-platinum (CIS) as first-line chemotherapy. A total of 50 samples from 102 patients have been successfully cultured and screened for in vitro chemosensitivity to 7 drugs, including CIS. The malignant nature of cells growing in culture was confirmed using a combination of karyology, morphology and immunohistochemical staining with HMFG2. Tumours were graded as sensitive (less than 40% of control 3H-leucine incorporation), intermediate (41-60% of control) or resistant (greater than 61% of control) to CIS. Correlation of in vitro sensitivity to cis-platinum with clinical response to cis-platinum assessed using CT scan and second-look laparotomy, showed positive correlation in 9/11 (89%) patients (8 = S/S; 1 = R/R); positive correlation between in vitro sensitivity to phosphoramide mustard and clinical response was also found in 4/6 patients receiving cyclophosphamide (3 = S/S; 1 = R/R). All patients with sensitive tumours showed a clinical response to cis-platinum. Comparison of cis-platinum sensitivity with sensitivity to phosphoramide mustard and melphalan showed that some tumours were sensitive only to cis-platinum; resistance to cis-platinum and sensitivity to phosphoramide mustard/melphalan was an infrequent occurrence. Some tumours which were resistant to cis-platinum showed sensitivity to adriamycin and bleomycin, particularly those from untreated patients. Sensitivity to 5-fluorouracil and resistance to cis-platinum was found in approximately equal proportions of tumours in both the treated and untreated groups.


Br. J. Cancer (1987), 56, 763–773 © The Macmillan Press Ltd., 1987

### *Cis*-platinum and ovarian carcinoma. *In vitro* chemosensitivity of cultured tumour cells from patients receiving high dose *cis*-platinum as first line treatment

A.P. Wilson[1], C.H.J. Ford[2], C.E. Newman[3] & A. Howell[4]

[1]*Oncology Research Laboratory, Derby City Hospital, Uttoxeter Road, Derby, DE3 3NE, UK;* [2]*The Newfoundland Cancer Treatment and Research Foundation, St. John's, Newfoundland;* [3]*Pfizer Canada Inc., PO Box 800, Pointe Claire, Dorval, Quebec, H9R AV2, Canada; and* [4]*Department of Medical Oncology, Christie Hospital and Holt Radium Institute, Manchester M20 9BX, UK.*

**Summary** A study on the *in vitro* sensitivity of tumour cells from patients with ovarian cancer has been carried out in parallel with a clinical study designed to evaluate the role of high-dose *cis*-platinum (CIS) as first-line chemotherapy. A total of 50 samples from 102 patients have been successfully cultured and screened for *in vitro* chemosensitivity to 7 drugs, including CIS. The malignant nature of cells growing in culture was confirmed using a combination of karyology, morphology and immunohistochemical staining with HMFG2. Tumours were graded as sensitive (<40% of control $^3$H-leucine incorporation), intermediate (41–60% of control) or resistant (>61% of control) to CIS.

Correlation of *in vitro* sensitivity to *cis*-platinum with clinical response to *cis*-platinum assessed using CT scan and second-look laparotomy, showed positive correlation in 9/11 (89%) patients (8=S/S; 1=R/R); positive correlation between *in vitro* sensitivity to phosphoramide mustard and clinical response was also found in 4/6 patients receiving cyclophosphamide (3=S/S; 1=R/R). All patients with sensitive tumours showed a clinical response to *cis*-platinum.

Comparison of *cis*-platinum sensitivity with sensitivity to phosphoramide mustard and melphalan showed that some tumours were sensitive only to *cis*-platinum; resistance to *cis*-platinum and sensitivity to phosphoramide mustard/melphalan was an infrequent occurrence. Some tumours which were resistant to *cis*-platinum showed sensitivity to adriamycin and bleomycin, particularly those from untreated patients. Sensitivity to 5-fluorouracil and resistance to *cis*-platinum was found in approximately equal proportions of tumours in both the treated and untreated groups.

Although response rates of approximately 50% have been achieved with alkylating agents in the treatment of ovarian cancer, survival times have not been significantly prolonged in this disease which still represents the leading cause of death from gynaecological cancer in the UK. New chemotherapeutic regimes are under continual evaluation and the finding that *cis*-platinum II diammine dichloride (CIS) could produce response rates of 30–40% in heavily pre-treated patients (Wiltshaw & Kroner, 1976; Bruckner *et al.*, 1978) argued for its trial as a first-line drug in the treatment of ovarian cancer. At the time this study was started there were no previous reports which described the use of high dose CIS (100 mg m$^{-2}$) in untreated patients with ovarian carcinoma, and a clinical trial was initiated to evaluate this regime. In conjunction with this, a laboratory study was set up to evaluate the *in vitro* response of tumour cells from trial patients to CIS. The patient group was potentially well suited to such a study because (a) most patients received a single drug, (b) there was surgical evidence of tumour burden at the onset of chemotherapy and (c) stringent response criteria including the use of computerised tomography and second look laparotomies (2LL) were used to evaluate response.

Results obtained with *in vitro* chemosensitivity testing of human ovarian tumour cells growing as a monolayer in microtitration plates have previously been described (Wilson & Neal, 1981). More recent studies using cell lines have shown that clonogenic assays and the microtitration assay give similar chemosensitivity results in spite of their intrinsic differences (Wilson *et al.*, 1984). The microtitration assay was chosen for this study in preference to the clonogenic assay used by other groups, (e.g. Hamburger *et al.*, 1978; Courtenay & Mills, 1978; Von Hoff *et al.*, 1983; Simmonds & McDonald, 1984) because of the low cell requirement which offers the facility of multiple drug screening over several drug concentrations.

The aims of the study were (i) to compare *in vitro* sensitivity to CIS with *in vivo* response to single agent therapy with this drug; (ii) to evaluate *in vitro* sensitivity to CIS after completion of chemotherapy and (iii) to compare *in vitro* sensitivity to CIS with sensitivity to other drugs in tumours from untreated and treated patients. Attention has also been focussed on the critical aspects specific to the use of monolayer cultures, including cell identification and the effect of stromal cell contamination on chemosensitivity profiles.

### Materials and methods

### Patients

Tumour samples were obtained from 72 patients in Birmingham and 30 patients in Manchester, all with histologically proven ovarian malignancy. Specimens comprising solid tumours, ascitic and pleural fluids and peritoneal washings were obtained from staging laparotomies, second look laparotomies and paracenteses.

### Chemotherapy

Birmingham patients were mainly treated with 5 courses of 100 mg m$^{-2}$ CIS, given as a bolus i.v. injection at three weekly intervals; a separate patient group was treated with a combination of CIS (50 mg m$^{-2}$), adriamycin – ADM (50 mg) and cyclophosphamide – CYM (1 gm) (CAP). In Manchester, treatment protocols comprised either 3 courses of CIS (100 mg m$^{-2}$) at three weekly intervals with 15 mg bleomycin – BLM administered i.v. at weekly intervals, followed by consolidation with at least 5 courses of CYM (1 gm$^{-2}$), or CYM (1 gm$^{-2}$) at 3 weekly intervals for 10 courses; a separate patient group received CIS (80 mg m$^{-2}$)

Correspondence: A.P. Wilson.
Received 20 January 1987; and in revised form, 23 June 1987.

and ADM ($60\,mg\,m^{-2}$) for 6 courses at 3 to 4 weekly intervals.

*Response evaluation*

A complete clinical response was defined by the disappearance of all signs of disease, and a partial response by an approximate reduction of 50% in tumour masses. Computerised tomography was used to assist in clinical evaluation. A complete surgical response was defined as the disappearance of all macroscopic disease at second look laparotomy, and a partial surgical response as a measured reduction of 50% in the largest mass present at staging laparotomy.

*Cell preparation*

Methods which have been described previously (Wilson & Neal, 1981) were used with the following modifications: disaggregation of solid tumour tissue was carried out using $2\text{–}4\,mg\,ml^{-1}$ collagenase (Worthington) and $50\,\mu g\,ml^{-1}$ DNA-ase (Miles Chemical Co.) in growth medium – (GM – Dulbecco's Modification of Eagles Medium supplemented with 20% foetal calf serum, 1 mM glutamine, 1 mM sodium pyruvate, $20\,IU\,l^{-1}$ insulin, $20\,IU\,ml^{-1}$ penicillin, $20\,\mu g\,ml^{-1}$ streptomycin and $3.7\,g\,l^{-1}$ sodium bicarbonate). After washing, disaggregated cells were adjusted to a final concentration of $10^5$ viable cells $ml^{-1}$ in GM. Viabilities of >90% were routinely obtained. Flat bottomed microtitration plates (Nunc) were seeded with $200\,\mu l$ of the cell suspension per well, and were then incubated at 37°C in an atmosphere of 95% air/5% $CO_2$. In some specimens cultures were initiated in $75\,cm^2$ plastic culture flasks and passage 1 cells were used for the assay. Cells from some tumours would not adhere to plastic and when this occurred the plates were centrifuged at $\sim 200\,g$ for 10 min prior to the removal of medium at each stage of the assay.

*Cell identification*

The presence of tumour cells in the original cell suspension was confirmed by microscopic examination of smears stained with haematoxylin and eosin; histological diagnosis of ovarian malignancy was confirmed in the biopsy specimens. Slide chambers (Labtek) were routinely set up for all samples and used for the identification of cell types growing in the monolayer. Cultures were fixed in acetic acid:methanol (1:3 i.v./v) and stained with haematoxylin and eosin. Conventional criteria as defined in the literature (Ioachim *et al.*, 1974; Whitehead & Hughes, 1975; Mouriquand *et al.*, 1978) were used to identify putative mesothelial cells, fibroblasts and tumour cells. Additionally, the same slide chamber cultures were destained with acid-alcohol and alcohol and subsequently used for immunohistochemical staining with OC125 (Bast *et al.*, 1981) and HMFG2 (Taylor–Papadimitriou *et al.*, 1981), using an indirect immunoperoxidase technique to confirm the presence of epithelial cells. Chromosome preparations were also made of some cultures using routine techniques.

*Drugs*

The following drugs were tested: adriamycin – ADM (Farmitalia, Carlo Erba Ltd.), bleomycin – BLM (Lundbeck Ltd.), *cis*-platinum II diammine dichloride – CIS (NCI, Bethesda), 5-fluorouracil – 5-FU (Roche Products Ltd.), melphalan – MEL (Burroughs Wellcome Ltd.) and vinblastine – VLB (Velbe, Eli Lilly). The concentrations used were selected to include achievable plasma levels (Alberts & Chen, 1980). ADM was tested at 2, 0.2 and $0.02\,\mu g\,ml^{-1}$, VLB at 1, 0.1 and $0.01\,\mu g\,ml^{-1}$ and all other compounds at 10, 1 and $0.1\,\mu g\,ml^{-1}$. In 9 tumour cultures CIS was tested over an extended concentration range (10, 5, 3, 2, 1, $0.1\,\mu g\,ml^{-1}$). MEL was dissolved in dimethyl sulphoxide (DMSO) prior to dilution in GM, and a solvent control at the corresponding dilutions was routinely included. Other compounds were dissolved in GM and all solutions were made up immediately before use, using aseptic technique without filtration.

*Cytotoxic determination*

Drugs were added 24–48 h after culture initiation and were left in situ for 48 h. Following a 24 h recovery period in GM only the amount of $^3$H-leucine incorporated into protein was determined using previously described methods (Freshney *et al.*, 1975). Results were expressed as a percentage of the mean control values of $^3$H-leucine incorporation. Standard deviations of the test means ($n=3$) were routinely $< \pm 10\%$ of the control mean ($n=3$), although higher values occasionally occurred. Reproducibility of the assay was assessed by comparing results from 25 different assays for chemosensitivity to CIS, using an ovarian tumour cell line, OAW 42 (passage 30–66). The mean percentage of control values ± standard deviation were 1.2% ± 0.8%, 26% ± 15% and 72% ± 23% at 10, 1 and $0.1\,\mu g\,ml^{-1}$ respectively.

*Stromal cell contamination*

Pure fibroblast or mesothelial cell cultures were obtained from 4 samples. The chemosensitivity profiles of these normal cell populations were measured for comparison with the tumour cells. In addition the effect of combining different proportions of both mesothelial cells and fibroblasts with the cell line OAW 42 on the measured chemosensitivity was determined. Proportions of 10%, 30%, 50%, 70% and 90% stromal cells with tumour cells were used as the initial cell innocula in the microtitration plate assay.

### Results

In Birmingham, 104 samples were obtained from 72 patients with ovarian malignancy. Culture of 92 samples was attempted and successful growth with <30% stromal cell contamination was achieved with 35 samples (39%) from 27 patients. These included multiple sites from 4 patients ($n=3$, $n=4$, $n=2$, $n=2$) and repeat samples from one patient ($n=2$). Of the tumours which grew successfully 26 were from untreated patients, (5 ascites, 21 solid tumours) and 9 from treated patients, including relapse and second-look laparotomies (4 ascites, 5 solid tumours). In Manchester, 38 samples were obtained from 30 patients and successful growth was achieved with 15 (39%). Of these 15 samples, 10 were from relapsed patients (10 ascites) and 5 were from untreated patients (2 solid tumours, 2 ascites, 1 pleural fluid). Repeat samples were grown successfully from one patient ($n=3$). Reasons for the failure to obtain adequate cultures with some samples included excessive stromal cell growth (>30%), inadequate growth of tumour cells, paucity of tumour sample, nature of tumour sample (e.g., necrotic, cystic, fibrotic), absence or very low yield of tumour cells in fluids and infection.

*Cell identification*

Preliminary staining of freshly fixed slide chamber cultures of 3 ovarian tumour cell lines, 8 mesothelial cell cultures and 2 fibroblast cultures was performed with OC 125 and HMFG2. Mesothelial cells and fibroblasts were weakly reactive with OC 125 whereas HMFG2 reacted only with the ovarian tumour cell lines. Subsequent staining of slide cultures for retrospective confirmation of cell type was, therefore, done with HMFG2. A total of 34 specimens were stained which, according to morphological criteria, included 9 stromal cell cultures, 7 pure tumour cell cultures and 18 mixed cell cultures containing varying proportions of normal cells and tumour cells. No stromal cell cultures stained with HMFG2 and putative tumour cells stained in all but 3

samples (64, 65, 11 M). Karyotyping of samples 64 and 65 confirmed the malignant nature of these cells. In sample 64 a mode of 44–48 was obtained in 52/52 spreads, all of which contained abnormal chromosomes. Samples 65 showed a mode of 42–46 in 64/88 spreads with chromosomal abnormalities which clearly distinguished them from the normal cells comprising 26% of the total population. Sample 11 M was a mixed homologous mesenchymal sarcoma of the ovary which showed a bizarre morphology without displaying any features typical of epithelial cells. The proportion of stromal cells present in cultures was thus based on a semi-quantitative assessment using morphology, HMFG2 staining and karyology.

*Effect of stromal cells on the chemosensitivity of OAW 42*

Four pure stromal cell cultures (2 fibroblast, 2 mesothelial) were screened for chemosensitivity. The results summarized in Table I show that these populations were comparatively chemoresistant. The effect of introducing increasing proportions of normal cells into the tumour cell population (OAW 42) on sensitivity to CIS is shown in Figure 1a, b for fibroblasts and mesothelial cells. It is apparent that the decrease in sensitivity to CIS was proportional to the increase in normal cell contamination. At 30% contamination the decrease in sensitivity was 7% at $1 \mu g ml^{-1}$ of CIS, and this was within the experimental variation (see **Materials and methods**) of the assay, and contrasts with a decrease in sensitivity of 30% at the 50% contamination level. Therefore, only those cultures which showed <30% stromal cell contamination have been used in the comparison between clinical data and *in vitro* data. The tumour cell cultures exhibited wide variations (which exceeded the experimental variation) in sensitivity to the different drugs tested and confirmed the earlier findings with the assay (Wilson & Neal, 1981) that *in vitro* chemosensitivity reflected *in vivo* response rates to single agent chemotherapy.

**Table I** Sensitivity to chemotherapeutic agents of fibroblasts and mesothelial cells from solid carcinoma and malignant effusions

| | Conc[N] $\mu g ml^{-1}$ | *Fibroblasts* 7[a] | *Fibroblasts* 53 | *Mesothelial cells* 53 | *Mesothelial cells* 16 |
|---|---|---|---|---|---|
| *ADM* | 2.0 | 1[b] | 104 | 48 | 4 |
| | 0.2 | 74 | 103 | 79 | 76 |
| | 0.02 | 98 | 100 | 89 | 70 |
| *CIS* | 10.0 | 27 | <1 | 1 | 2 |
| | 1.0 | 92 | 80 | 69 | 74 |
| | 0.1 | 105 | 89 | 87 | 77 |
| *PM* | 10.0 | 123 | 99 | 74 | – |
| | 1.0 | 117 | 98 | 89 | – |
| | 0.1 | 114 | 92 | 101 | – |
| *MEL* | 10.0 | 50 | – | 73 | – |
| | 1.0 | 103 | – | 86 | – |
| | 0.1 | 168 | – | 97 | – |

[a]Normal cell cultures were tested from patients 7, 53 and 16. Passage 1 cells were used for 7 and 16, and passage 3 for 53; [b]Percent of control $^3H$-leucine incorporation.

*5-FU, VLB, ADM, BLM, PM*

Dose response curves for these drugs were similar to those presented previously (Wilson & Neal, 1981) and will not, therefore be shown for individual patients. Data from the curves are summarised in Table II with treated and untreated patients shown separately. The largest variation in sensitivity between tumours from individual patients occurred at $10 \mu g ml^{-1}$ of PM, and for 5-FU, VLB, ADM and BLM there was considerable variation at the two higher

**Figure 1** The effect of introducing varying proportions of fibroblasts (A) and mesothelial cells (B) on the measured dose-response of an ovarian cell line (OAW 42) to *cis*-platinum.
● – 100% stromal cells (fibroblasts or mesothelial cells)
○ – 100% OAW 42
◇ – 10% stromal cells
□ – 30% stromal cells
■ – 50% stromal cells
◆ – 70% stromal cells
◉ – 90% stromal cells concentrations tested. There was no significant difference in values from tumours of treated *versus* untreated patients with 5-FU, BLM or ADM. There was a trend towards increased sensitivity to VLB in the treated group (43±28% *vs.* 55±34%) and to decreased sensitivity to PM in the treated group (77±38% *vs.* 58±35%), although these differences did not reach significance (Students *t* test: $P=0.2$, $P=0.1$ respectively).

*CIS and MEL*

Dose response curves for these drugs tested against human ovarian tumour cells have not been presented previously using this assay, and they are shown in Figure 2a, b and 3a, b. Summaries of the data are shown in Table III.

Results obtained when DMSO was tested alone at dilutions of 1/100, 1/1000 and 1/10,000 showed that the compound had significant activity against some tumours at a

**Table II** Summary of % of control $^3$H-leucine incorporation for PM, 5-FU, ADM, BLM and VLB

| | | | | | | |
|---|---|---|---|---|---|---|
| *PM* | *10*[b] | | *1.0*[b] | | *0.1*[b] | |
| | *U* | *T* | *U* | *T* | *U* | *T* |
| x±s.d. | 58±13 | 77±38 | 80±31 | 101±44 | 92±22 | 115±61 |
| range | 1–128 | 20–188 | 19–136 | 64–262 | 58–162 | 71–285 |
| % sensitive[a] | 39 | 21 | 19 | 0 | 0 | 0 |
| *5-FU* | *10* | | *1.0* | | *0.1* | |
| | *U* | *T* | *U* | *T* | *U* | *T* |
| x±s.d. | 29±18 | 30±22 | 52±22 | 51±30 | 77±24 | 87±42 |
| range | 8–51 | 3–73 | 16–93 | 16–133 | 23–135 | 49–182 |
| % sensitive | 79 | 84 | 42 | 54 | 12 | 8 |
| *ADM* | *2* | | *0.2* | | *0.02* | |
| | *U* | *T* | *U* | *T* | *U* | *T* |
| x±s.d. | 24±27 | 20±23 | 77±27 | 72±32 | 87±16 | 94±36 |
| range | 1–105 | 1–72 | 33–165 | 18–136 | 53–122 | 45–210 |
| % sensitive | 78 | 82 | 7 | 9 | 0 | 0 |
| *BLM* | *10* | | *1.0* | | *0.1* | |
| | *U* | *T* | *U* | *T* | *U* | *T* |
| x±s.d. | 46±31 | 46±24 | 69±33 | 64±23 | 81±23 | 83±20 |
| range | 7–114 | 16–104 | 20–174 | 29–116 | 50–165 | 44–115 |
| % sensitive | 56 | 56 | 32 | 25 | 4 | 12 |
| *VLB* | *1* | | *0.1* | | *0.01* | |
| | *U* | *T* | *U* | *T* | *U* | *T* |
| x±s.d. | 55±34 | 43±28 | 60±36 | 54±29 | 71±32 | 63±29 |
| range | 11–180 | 13–112 | 15–188 | 14–119 | 17–106 | 20–112 |
| % sensitive | 50 | 69 | 46 | 61 | 25 | 38 |

[a]Sensitive – <50% of control; [b]drug concentration in $\mu$g ml$^{-1}$; U = untreated patients; T = treated patients; PM – 31 U, 19 T; 5-FU – 24 U, 13 T; ADM – 27 U, 11 T; BLM – 25 U, 16 T; VLB – 24 U, 13 T.

**Table III** Summary of % of control $^3$H-leucine incorporation for CIS and MEL

| | | | | | | |
|---|---|---|---|---|---|---|
| *CIS* | *10*[a] | | *1.0*[a] | | *0.1*[a] | |
| | *U* | *T* | *U* | *T* | *U* | *T* |
| x±s.d. | 6±13 | 11±18 | 47±23 | 70±40 | 83±18 | 101±48 |
| range | 1–58 | 1–73 | 2–113 | 10–137 | 38–128 | 54–236 |
| % sensitive | 97 | 95 | 52 | 37 | 10 | 0 |
| *MEL* | *10* | | *1.0* | | *0.1* | |
| | *U* | *T* | *U* | *T* | *U* | *T* |
| x±s.d. | 58±23 | 65±34 | 82±24 | 85±25 | 95±17 | 90±11 |
| range | 11–101 | 14–143 | 14–107 | 42–123 | 65–121 | 69–105 |
| % sensitive | 33 | 36 | 5 | ·9 | 0 | 0 |

[a]Drug concentration in $\mu$g ml$^{-1}$; Sensitive <50% of control; U = untreated patients; T = treated patients; CIS – 31 U, 19 T; MEL – 21 U, 11 T.

dilution of 1/100. It was routinely included in 34 assays and in 26 of these the percentage of control values ranged from 72–100%, whilst for another 5 tumours (MEL resistant) there was stimulation to >110% (maximum 281%). For 3 other tumours (MEL sensitive) values of 57%, 44% and 64% were obtained and these have been excluded from the data presented in Figure 2a, b and summarised in Table III. No tumours showed marked sensitivity to MEL at 10 $\mu$g ml$^{-1}$, the concentration which elicited greatest variation between tumours, and, although there was a slight shift towards resistance in the treated group, this value did not approach significance.

CIS was routinely tested at 0.1, 1.0 and 10 $\mu$g ml$^{-1}$ against 50 tumours (31 untreated, 19 treated) (Figure 3a, b) and additional concentrations were included for 9 tumours (Figure 4). For both treated and untreated tumours the widest range of sensitivities was observed at 1 $\mu$g ml$^{-1}$ and the difference between untreated and treated tumours (47% ± 23% *vs.* 70% ± 40%) was significant (unpaired Students '*t*' test, $2P = 0.05$). Inclusion of additional concentrations between 1 and 10 $\mu$g ml$^{-1}$ (Figure 4) did not alter the relative sensitivity ratings of 8 of the tumours, but one (40 M) did show a marked increase in sensitivity between 1 and 2 $\mu$g ml$^{-1}$.

*Comparison of* in vitro *results with clinical outcome to chemotherapy*

Correlations between *in vitro* results and *in vivo* response to treatment were possible in 17 untreated patients and 14 treated patients. Relevant data are presented in Tables IV and V for each group. Ten untreated patients received CIS only (Section A in Table IV) and the clinical response rate in this group was 80%. In the absence of defined response criteria for this assay, cut-off points for CIS and PM were chosen to give the highest degree of correlation between *in vitro* data and clinical outcome. Accordingly, the following criteria were used at 1 $\mu$g ml$^{-1}$ of CIS: – sensitive – <40% of control; intermediate – 41–60% of control; resistant – >61% of control. There were 5 patients in the sensitive group (56, 53, 20, 8, 9 M) all of whom responded regardless of tumour burden at the onset of chemotherapy. There were 4 patients in the intermediate group (74, 66, 78, 53-multiple sites received from 53), 3 of whom responded to treatment. One of these patients also had sensitive tumour cells (53), one had a complete pelvic clearance at staging laparotomy (78) and one was found to have residual disease at second look laparotomy (66). The non-responder was a stage IV patient with liver involvement (74). In the resistant group

**Figure 2** Dose-responses of tumour cells from untreated (A) and treated (B) patients to melphalan.

**Figure 3** Dose-responses of tumour cells from untreated (A) and treated (B) patients to *cis*-platinum.

**Figure 4** Dose-responses of tumour cells from one untreated patient (41 M) and 9 treated patients to additional concentrations of CIS between 1 and $10\mu g ml^{-1}$.

there were 2 patients (65, 26) one of whom responded to treatment (65) and one of whom had progressive disease (26). The responder was Figo, stage I and had minimal residual disease at the onset of chemotherapy, whilst the patient who progressed was again Stage IV with liver involvement. In this group of 11 patients *in vitro* and *in vivo* results correlated for 9/11 (89%) patients (8=S/S; 1=S/R; 1=R/S). Six previously untreated patients received CYM only (Section B, Table IV). For the purpose of comparison of *in vitro* data with clinical outcome, the following criteria were used at $10\mu g ml^{-1}$ of PM: – sensitive – <50% of control; intermediate – 51–60% of control; resistant – >61% of control. Accordingly, there were 2 patients in the sensitive group (9 M, 3 M) both of whom had partial responses (9 M was included in the untreated CYM group because she had responded to 3 courses of CIS but treatment had been changed to CYM because of impaired renal function). There were also 2 patients in each of the intermediate (34 M, 41 M) and resistant (62, 11 M) groups. In each of the latter groups there was one partial responder and one progressive disease. In the intermediate group the patient with PD was stage IV (34 M). In the resistant group the patient who responded had a mixed homologous mesenchymal sarcoma of the ovary, a tumour which contains both sarcomatous and adenocarcinomatous elements. The cells which grew were obviously bizarre, but did not stain with HMFG2 and did not show typical epithelial morphology. They were, therefore, believed to represent the sarcomatous component of the tumour and lack of correlation in this instance could be attributed to histological heterogeneity. For these 6 patients *in vitro* and *in vivo* results correlated in 4/6 cases (3=S/S; 1=R/R; 1=S/R; 1=R/S). Two other patients received mixed chemotherapy (Section C, Table IV), one of whom was resistant *in vitro* and *in vivo* and failed to respond to treatment (59), and the other patient had a partial response to CIS/ADM to which drugs her tumour cells showed intermediate sensitivity and sensitivity respectively (28 M).

In the treated group (Table V) there were 10 patients who had received CIS at some time; 5 of these had received CIS only as first line therapy (28, 42, 43, 32 M, 40 M), one had received CIS in combination (CAP) (73) and 4 had received CIS as second-line therapy, either alone (40, 48/89, 25 M) or in combination (30 M – CAP). The specimens included ascites from relapse patients (48/49, 28, 42, 43, 25M, 30 M, 32 M, 40 M) and samples from 2LLs (40, 73). Both the samples from 2LL were of intermediate sensitivity. Of the two relapse samples of intermediate sensitivity, one came from a stage IV patient with progressive disease (28) and the other from a patient who had a complete response to first-line chemotherapy with CIS (42) (see 8 in Table IV). In the resistant group 43 and 32 M both had a PR to first-line chemotherapy with CIS; 40 M had had 13 courses of CIS/ADM and relapsed whilst receiving chemotherapy; 25 M relapsed on CYM/FU/MTX and failed to respond to CIS and 30 M failed to respond initially to treosulfan and subsequently to CAP. The tumour cells of one patient showed exquisite sensitivity to CIS (48/49), although she was in relapse following treatment with this drug. However, throughout chemotherapy ascites had been completely controlled, but there was subjective evidence of increase in size of a mass in the Pouch of Douglas leading to cessation of treatment.

Eight patients had received CYM at some time. Two of these were still sensitive (48/49, 38 who had received low-dose CYM for 3 months) and 6 were resistant. All of the resistant group had had some response to treatment with CYM but had relapsed during treatment.

### *Comparison of multiple sites*

More than one tumour sample was tested from each of four patients. These were 20 (R.ovary and ascites), 40 (rectum, R.ovary, omentum), 53 (R.ovary, L.ovary, omentum, ascites) and 59 (L.ovary, R.ovary). For 40 and 59 similar chemosensitivity profiles were obtained for all sites tested. There were differences between sites for 20 and 53 and dose response curves are shown in Figure 5 and 6. Cells from the ascites (20, 53) and omentum (53) were more chemosensitive than their solid counterparts to some of the drugs which were tested. Ascitic cells from 20 were much more sensitive to 5-FU and slightly more sensitive to ADM and PM than the solid tumour cells. Both populations were of equal sensitivity to CIS. With 53 the omental and ascitic cells were markedly more sensitive to ADM, CIS and VLB, and slightly more sensitive to PM, BLM and 5-FU than cells from the solid tumours.

### *Comparison between CIS sensitivity and sensitivity to other drugs*

Table VI shows a comparison between tumours showing sensitivity or resistance to CIS and the sensitivity of the same tumours to other drugs. Thus, in the untreated group, there were 16 CIS-sensitive tumours of which 10 were also sensitive to PM, whilst only 2/15 tumours which were resistant to CIS still showed some sensitivity to PM. In the treated group 4/7 CIS-sensitive tumours were also sensitive to PM but no CIS-resistant tumours showed sensitivity to PM. A similar distribution was observed with MEL in the untreated group, but in the treated group cross-sensitivity and cross-resistance was less clearly defined. In the untreated group other drugs which showed a trend towards cross-sensitivity and cross-resistance with CIS were BLM at $1\mu g ml^{-1}$ (6/13 *vs.* 2/13) and 5-FU at $1\mu g ml^{-1}$ (8/14 *vs.* 2/10). Sensitivity to the higher concentrations of ADM, BLM, 5-FU and also to VLB was approximately similarly distributed between CIS sensitivity and resistance. In the treated group cross-sensitivity and cross-resistance was more marked for ADM and BLM but was unchanged for 5-FU

**Table IV** Correlations between *in vitro* sensitivity and clinical response in untreated patients

| *Lab. no.* | *Figo* | *Disease status after surgery* | *Chemotherapy* | *Response Clinical* | *Response Surgical* | In vitro response | *Correlation* |
|---|---|---|---|---|---|---|---|
| *A* | | | | | | | |
| 8 | III | inoperable | CIS × 5 | CR | PR(CR)+ | 23 ± 4% | S/S |
| 20 | III | > 2 cm | CIS × 5 | CR | PR(CR)+ | 17 ± 3% | S/S |
| 53 | | | CIS × 5 | CR | PR | 54 ± 11% (solid) | I/S |
| | | | | | | 27 ± 8% (ascites) | S/S |
| 56 | | < 1 cm | CIS × 5 | CR | CR(+) | 36 ± 6% | S/S |
| 9 M | IV | GRD | CIS × 3 CYM (renal failure) | PR | ND | 40 ± 4% | S/S |
| 66 | III | > 2 cm | CIS × 5 | CR | PR | 59 ± 2% | I/S |
| 74 | IV | 4 cm lesion liver invol. | CIS × 2 | PD | – | 41 ± 7% | I/R |
| 78 | III | clearance | CIS × 5 | CR | CR | 52 ± 6% | I/S |
| 26 | IV | liver invol. | CIS | PD | – | 65 ± 11% | R/R |
| 65 | I | clearance | CIS × 5 | CR | ND | 63 ± 8% | R/S |
| *B* | | | | | | | |
| 3 M | III | inoperable | CYM × 2 | PR | (died of coronary) | 39 ± 18% | S/S |
| 9 M | IV | GRD | CIS × 3 CYM | PR | stable disease | 48 ± 6% | S/S |
| 34 M | IV | GRD < 2 cm | CYM | PD | ND | 55% | I/R |
| 41 M | III | GRD | CYM | PR | ND | 56% | I/S |
| 62 | | | CYM | SD | ND | 67% | R/R |
| 11 M | III | GRD | CYM | PR | ND | 123% | R/S |
| *C* | | | | | | | |
| 59 | III | GRD > 2 cm | CMB, CYM MTX | PD | ND | PM - 74% MTX - 76% CIS - 26% | R/R |
| 28 M | III | GRD | CIS, ADM | PR | ND | CIS - 60% | R/S |
| | | | | | | ADM - 7% | S/S |

*Disease status:* GRD = gross residual disease; MRD = minimal residual disease; *Response:* CR = complete response; PR = partial response; PD = progressive disease; SD = stable disease; *Surgical response:* (CR) indicates surgical conversion from PR→CR. + indicates microscopic disease. ND = not done; *Correlation:* CIS: S = < 40% of control (sensitive); I = 41–60% of control (intermediate); R = > 61% of control (resistant); PM: S = < 50% of control (sensitive); I = 51–60% of control (intermediate); R = > 61% of control (resistant).

**Figure 5** A comparison of dose-responses of tumour cells from solid tumour (●——●) and ascites (○——○) of the same patient (OAW 20). All drug concentrations (horizontal axes) are in $\mu g\,ml^{-1}$. ADM = adriamycin, CIS = *cis*-platinum; PM = phosphoramide mustard; 5-FU = 5-fluorouracil. Error bars = s.d.

**Table V** *In vitro* response data for treated patients

| *Lab. no.* | *Previous chemotherapy* | In vitro *response* | *Comments* |
|---|---|---|---|
| 48/49 | CYM; CIS x 4; CMB; BLM. | CIS–10%, CMB–24%, PM–20%, BLM–28%. | One year's treatment with endoxana; pleural effusion and mass in Pouch of Douglas. 4 courses of CIS given, during which ascites completely controlled. Chemotherapy stopped because increase in size of mass in Pouch of Douglas. |
| 28 | CIS; MEL. | CIS–46%, MEL–40%. | Progressive disease on first-line chemotherapy with CIS. |
| 40 | TREO; CIS. | Right CIS–41%, TREO–59%. Omentum CIS–44%, TREO–67%. Rectum CIS–50%, TREO–71%. | Patient was GRD inoperable; good response to TREO obtained. CIS given after relapse but no evidence of regression: patient converted to PR at 2LL. |
| 42 | CIS x 5. | CIS–55%. | See Lab. No. 8 in **Table IV**; some resistance to CIS developed. |
| 73 | CIS; ADM; CYM. (=CAP x 6). | CIS–43%, PM–64%, ADM–24%. | Clinically assessed as CR. 2LL revealed widespread tumour deposits. |
| 43 | CIS. | CIS–67%. | PR obtained to first-line chemotherapy with CIS. |
| 25 M | CYM, 5-FU, MTX– relapse. CIS x 3. | CIS–175%, PM–188%. | Relapsed on CYM/5-FU/MTX. PD after 3 courses of CIS. |
| 30 M | TREO 4/12; CIS; ADM; CYM (CAP). | CIS–110%, ADM–35%, PM–105%. | PD on treosulfan after 4/12 treatment; no reponse obtained to CAP. |
| 32 M (=9 M **Table IV**) | CIS x 3; BLM x 3; CYM. | CIS–91%, BLM–71%, PM–102%. | Post operative residual disease with bowel involvement. PR on CIS/BLM consolidated with CYM because of renal failure. 17 month survival w/o remission. |
| 40 M | CIS/ADM; CMB; MEL. | CIS–66%, PM–82%, ADM–62%. | Metastatic disease Stage IV. CIS/ADM x 13; CMB x 1; MEL x 1. |
| 31 M | CYM x 5; MEL. | CIS–137%, PM–147%, MEL–131%. | PD on CYM and MEL. |
| 12 M | CYM x 6. | CYM–63%, (CIS–70%). | Two months of PR obtained. |
| 38 | low dose CYM x 3. | PM–48%. | 2LL after three months on low dose CYM. |
| 1 M | MEL. | MEL–67%, (CIS–75%). | Relapse after two years on Melphalan. |

**Figure 6** A comparison of dose-responses of tumour cells from 4 sites in the same patient (OAW 53). ● = right ovary; ○ = left ovary; ■ = omentum; □ = ascites. All drug concentrations (horizontal axes) are in $\mu g\,ml^{-1}$. BLM = bleomycin; VLB = vinblastine. Error bars, which have been omitted for clarity, were of the same order of magnitude as those shown in **Figure 5**.

**Table VI** Comparison between CIS sensitivity and sensitivity to other drugs

| | *PM* | *MEL* | *ADM* | | *BLM* | | *5-FU* | | *VLB* |
|---|---|---|---|---|---|---|---|---|---|
| Drug concentration | 10[a] | 10 | 2.0 | 0.2 | 10 | 1.0 | 10 | 1.0 | 0.1 |
| *Untreated patients* | | | | | | | | | |
| Sensitive to CIS | 10/16[b] | 6/9 | 13/15 | 3/15 | 9/13 | 6/13 | 11/14 | 8/14 | 7/12 |
| Resistant to CIS | 2/15 | 1/12 | 8/12 | 1/12 | 5/12 | 2/13 | 8/10 | 2/10 | 5/12 |
| *Treated patients* | | | | | | | | | |
| Sensitive to CIS | 4/7 | 2/5 | 6/6 | 1/6 | 6/7 | 2/7 | 6/7 | 5/7 | 4/7 |
| Resistant to CIS | 0/12 | 2/6 | 3/5 | 1/5 | 3/9 | 2/9 | 5/6 | 2/6 | 5/6 |

[a]Drug concentrations are in $\mu g\,ml^{-1}$; [b]i.e., 10/16 tumours which were sensitive to CIS were also sensitive to PM.

with 6/7 CIS-sensitive tumours and 5/6 CIS-resistant tumours showing sensitivity to 5-FU. With VLB there appeared to be an association between resistance to CIS and increased sensitivity to VLB.

### Discussion

The results obtained with the microtitration assay confirm and extend previous findings (Wilson & Neal, 1981), *viz.* that it can be used to show variations in the chemosensitivities of human ovarian tumours which reflect the clinically found pattern of response to chemotherapy.

The overall success rate with the assay was ~40% which is considerably lower than the ~80% success rate which has been reported by groups using the clonogenic assay (Von Hoff *et al.*, 1983; Simmonds & McDonald, 1984). However, the number of drug tests achieved for each successful monolayer culture exceeds that which is normally possible for the clonogenic assay. Poor growth in monolayer and/or non-adherence of tumour cells contributed, in part, to the reduced success rate, but recent data describing the use of extracellular matrix to improve the monolayer growth of human ovarian tumour cells (Baker *et al.*, 1986) indicates that the number of successful cultures may be improved by appropriate modification of culture conditions. Stromal cell overgrowth was also a problem and the necessity for adequate cell identification cannot be over-emphasised. Whilst there appeared to be no interaction in monolayer between stromal cells (fibroblasts and mesothelial cells) and tumour cells with the particular cell line used, in that increasing numbers of drug-resistant stromal cells produced a proportional decrease in drug sensitivity of the total mixed population (see Figure 1a, b), the possibility that this may occur with other tumour cells cannot be excluded. The degree of stromal cell contamination (from 0–30%) did not influence the presence or absence of positive correlation in those tumour cultures for which *in vivo* and *in vitro* data was available, which indicates that this is an acceptable stromal contamination level for primary cultures.

The comparatively low levels of cell kill which were achieved with both PM and MEL perhaps reflects the short duration of the assay, which does not permit expression of delayed cytotoxicity (Freshney *et al.*, 1975). Certainly, higher levels of cell kill are achieved in the clonogenic assay with only a one hour exposure. The predictive accuracy of the microtitration assay may be improved with modifications permitting the use of a longer recovery period. Recent data also indicates that PM is an inappropriate metabolite of CYM to use for *in vitro* testing and that 4-hydroperoxy-cyclophosphamide is more relevant (Powers & Sladek, 1983). The high levels of leucine and glutamine which are present in growth medium are likely to impair the uptake of MEL (Vistica *et al.*, 1981) thus providing a further possible explanation for the low levels of cell kill in monolayer assay, contrasting with the clonogenic assay in which drug exposure is usually carried out in a balanced salt solution.

Although tumour cells displayed a greater sensitivity to CIS than they did to either PM or MEL, this was achieved at higher concentrations of CIS than those used in the clonogenic assays, which, taken in conjunction with the long drug exposure time, indicates reduced sensitivity of the monolayer assay. Although the theoretical difference in exposure time is 47 h, the actual difference is much less than this due to the instability of the drug solution. Pre-incubation of drug solutions has shown that there is a measurable decrease in activity of CIS by 12 h (6% of control compared with 23% of control at $1\,\mu g\,ml^{-1}$) and that activity is completely lost by 48 h (Wilson, unpublished data). The short recovery period also contributes to the reduced sensitivity of the assay and a prolonged recovery period of 12 days has also been shown to enhance the sensitivity of the assay (Wilson, unpublished data). In the monolayer assay described here 39% of all tumours tested were very sensitive to CIS (<40% of control) and 71% showed some sensitivity (<60% of control). These values are very similar to those reported by Simmonds and McDonald (1984) for CIS sensitivity (48% sensitive, 79% intermediate + sensitive). Thus, in spite of their intrinsic differences the monolayer and clonogenic assays are predicting similar response rates of primary ovarian carcinoma cells to CIS.

Correlation between *in vitro* sensitivity and clinical outcome in untreated patients was positive in 9/11 patients (8 = S/S; 1 = R/R) (89%). Correlation with extreme sensitivity to CIS (<40% of control) was very good with no false positives. The overall response rate was very high, however, since patients were previously untreated and received aggressive first-line chemotherapy. This contrasts with other studies in which patients have either been heavily pretreated or receiving a wide range of drugs. On the basis of the argument first put forward by Berenbaum (1974) a random prediction of 70% *in vitro* sensitivity in a patient group with an 80% response rate would give an overall positive correlation rate of 58% (56% = S/S; 2% = R/R), a value which is far exceeded by the 89% correlation rate obtained. The intermediate group presented a different picture, however, and there is some evidence to suggest that tumour burden plays an important role in the clinical outcome of this group. Patients with MRD were disease-free on completion of chemotherapy (~6 months) and, although it could be argued that there was no macroscopic disease present when chemotherapy was started, microscopic disease could be expected to have been present which, in the resistant or untreated patient, is likely to have manifested itself as gross disease in the 6 months following laparotomy. The observation that no stage IV patients with liver involvement and intermediate sensitivity tumours showed a clinical response provides the converse argument.

*In vitro* survival of >60% of control correlated well with clinical resistance in the treated group of patients. A finding of particular interest was the low level of resistance shown by tumour cells from patients who had received CIS and responded. Thus, tumours from patients 42, 43, and 73

showed values of 55%, 67% and 43% of control respectively, and 48/49 had cells which were very sensitive to CIS (<10% of control). All of these patients had had some response to CIS and, although one would not expect such a high level of sensitivity in 48/49, ascites in this patient was completely controlled whilst she was receiving the drug. In view of the difference in chemosensitivity exhibited by solid *vs.* ascitic tumour cells, the apparent lack of correlation may be attributable to this factor.

Extreme resistance to CIS (75% - 1 M; 175% - 25 M; 110% - 30 M; 137% - 31 M; 91% - 32 M) was seen only in some of the heavily pre-treated Manchester patients. The finding that tumours from responding patients who had received CIS showed lower levels of resistance either immediately after completion of chemotherapy or on relapse, suggests that high-dose CIS as first-line therapy does not induce extreme chemoresistance. This contrasts with the previously used palliative chemotherapy which invariably led to clinical resistance since therapy was continued to the point of relapse rather than to the point of limiting toxicity as is the case with CIS. There is clinical data to support the view that responses can be obtained following first-line therapy with CIS. Ozolls *et al.* (1987) reported that second-line responses to carboplatin could be obtained in patients who had responded to CIS and Sessa (1986) has shown that patients who responded to CIS could have a second response to iproplatin.

Comparison of *in vitro* sensitivity to CIS with *in vitro* sensitivity to other drugs showed that sensitivity to CIS did occur in the absence of cross-sensitivity to either PM or MEL. Sensitivity to PM or MEL and resistance to CIS was an infrequent occurrence. The absence of cross-sensitivity and cross-resistance for the high concentrations of ADM, BLM and 5-FU *vs.* CIS in the untreated group argues for the use of these drugs in combination with CIS. The increased incidence of cross-resistance to ADM and BLM in the treated group probably reflects the fact that these drugs were included in the chemotherapy which many patients received. The finding that cross-resistance to 5-FU did not develop in the treated group is of particular interest. The efficacy of this drug in the treatment of epithelial ovarian cancer has recently been demonstrated (Ozolls *et al.*, 1984) and it also has the advantage that it can be administered i.p. to achieve high therapeutic concentrations. The increased sensitivity to VLB which was found in treated patients reflects a similar observation made by Alberts (Alberts *et al.*, 1980) using the clonogenic assay. Unfortunately clinical application of this *in vitro* finding has not produced the expected response (Kavanagh *et al.*, 1984).

With the advent of effective combination chemotherapy regimens for the treatment of ovarian cancer the role of *in vitro* sensitivity testing in this disease is less clearly defined. In the past one of the main functions of predictive testing seems to have been to confirm resistance in the heavily pre-treated patient. This was of little therapeutic benefit since resistance to the majority of available drugs was already present. From the findings of the present study it is suggested that the assay could be of benefit in the following situations: (1) for stage IV patients who are likely to achieve a response to CIS if the tumour shows extreme *in vitro* sensitivity; (2) for patients with impaired renal function or other reasons for dose-reduction or chemotherapy other than CIS; (3) extreme *in vitro* sensitivity to CIS in untreated patients' tumours who subsequently receive the drug, respond and later relapse may be a good prognostic indication for obtaining a second-line response.

It is concluded that the microtitration assay provides *in vitro* information on the drug sensitivity of ovarian tumour cells which parallels the clinical response rates obtained with CIS and also those to be expected with single agent chemotherapy using other drugs (Wilson & Neal, 1981). With the increasing interest in the use of human tumour cell lines and primary cultures for screening new compounds the assay could be of use in this context.

The work was supported by a grant from the Medical Research Council and was carried out in the Department of Clinical Oncology, Queen Elizabeth Hospital, Birmingham and in the Department of Obstetrics and Gynaecology, Withington Hospital, Manchester. The support of Professor M. Elstein, in providing the latter facility, is gratefully acknowledged. Drugs were kindly supplied by the National Cancer Institute, Bethesda (CIS, PM); Farmitalia Carlo Erba Ltd. (ADM); Roche Products Ltd. (5-FU) and Burroughs Wellcome Ltd. (MEL). HMFG2 was kindly supplied by Dr J. Taylor-Papadimitriou and we thank Dr M. Moore, for assistance with the immunohistochemistry. We are indebted to all clinicians, ward staff and theatre staff who co-operated with the provision of tumour material, and also to Mrs S. Hood and Miss C. Rodgers of the Cytogenetics Department, East Birmingham Hospital for their work with the chromosome preparations. The expert technical assistance of Mrs B. Laher and Mrs C. Taylor is also gratefully acknowledged.

### References

ALBERTS, D.S. & GEORGE CHEN, H.S. (1980). In *Cloning of Human Tumour Stem Cells*, Salmon, S.E. (ed) Alan R. Liss Inc., N.Y., Appendix 4.

BAKER, F.L., SPITZER, G., AJANI, J.A. & 8 others (1986). Drug and radiation sensitivity measurements of successful primary monolayer culturing of human tumor cells using cell-adhesive matrix and supplemented medium. *Cancer Res.*, **46**, 1263.

BAST, R.C., FEENEY, M., LAZARUS, H., NADLER, L.M., COLVIN, R.B. & KNAPP, R.C. (1981). Reactivity of a monoclonal antibody with human ovarian carcinoma. *J. Clin. Invest.*, **68**, 1331.

BERENBAUM, M.C. (1974). Predicting response of human cancer to chemotherapy. *Lancet*, **ii**, 1141.

BRUCKNER, H.W., COHEN, C.J., WALLACH, R.C. & 5 others (1978). Treatment of advanced ovarian cancer with *cis*-dichlorodiammine platinum (II): Poor-risk patients with intensive prior therapy. *Cancer Treat. Rep.*, **62**, 555.

COURTNAY, V.D. & MILLS, J. (1978). An *in vitro* colony assay for human tumours grown in immune-suppressed mice and treated *in vivo* with cytotoxic agents. *Br. J. Cancer*, **37**, 261.

FRESHNEY, R.I., PAUL, J. & KANE, I.M. (1975). Assay of anti-cancer drugs in tissue culture; conditions affecting their ability to incorporate $^3$H-leucine after drug treatment. *Br. J. Cancer*, **31**, 89.

HAMBURGER, A.W., SALMON, S.E., KIM, M.B. & 4 others (1978). Direct cloning of human ovarian carcinoma cells in agar. *Cancer Res.*, **38**, 3438.

IOACHIM, H.L., SABBATH, M., ANDERSSON, B. & BARNER, H.R.K. (1974). Tissue cultures of ovarian carcinoma. *Lab. Invest.*, **31**, 381.

KAVANAGH, J.J., TAYLOR WHARTON, J. & RUTLEDGE, F.N. (1984). Continuous-infusion vinblastine for treatment of refractory epithelial carcinoma of the ovary: A Phase II trial. *Cancer Treat. Rep.*, **68**, 1417.

MOURIQUAND, J., MOURIQUAND, C., PETITPAS, E. & MERMET, M.A. (1978). Long-term tissue culture of human pleural effusions: A cytological follow-up. *In vitro*, **7**, 591.

OZOLLS, R.F., SPEYER, J.L., JENKINS, J. & MYERS, C.E. (1984). Phase II trial of 5-FU administered i.p. to patients with refractory ovarian cancer. *Cancer Treat. Rep.*, **68**, 1229.

OZOLLS, R.F., OSTCHEGA, Y., CURT, G. & YOUNG, R.C. (1987). High-dose carboplatin in refractory ovarian cancer patients. *J. Clin. Oncol.*, **5**, 197.

POWERS, J.F. & SLADEK, N.E. (1983). Cytotoxic activity relative to 4-hydroxycyclophosphamide and phosphoramide mustard concentrations in the plasma of cyclophosphamide treated rats. *Cancer Res.*, **43**, 1101.

SESSA, C. (1986). European studies with *cis* platin and *cis* platin analogues in advanced ovarian cancer. *Eur. J. Cancer Clin. Oncol.*, **22**, 1271.

SIMMONDS, A.P. & McDONALD, E.C. (1984). Ovarian carcinoma cells in culture: Assessment of drug sensitivity by clonogenic assay. *Br. J. Cancer*, **50**, 317.

TAYLOR-PAPADIMITRIOU, J., PETERSON, J.A., ARKLIE, J., BURCHELL, J., CERIANI, R.L. & BODMER, W.F. (1981). Monoclonal antibodies to epithelium-specific components of the human milk fat globule membrane: Production and reaction with cells in culture. *Int. J. Cancer*, **28**, 17.

VISTICA, D.T., VON HOFF, D.D. & TORAIN, B. (1981). Uptake of melphalan by human overian carcinoma cells and its relationship to the amino acid content of ascitic fluid. *Cancer Treat. Rep.*, **65**, 157.

VON HOFF, D.D., CLARK, G.M., STOGDILL, B.J. & 7 others (1983). Prospective clinical trial of a human tumour cloning system. *Cancer Res.*, **43**, 1926.

WHITEHEAD, R.H. & HUGHES, L.E. (1975). Tissue culture studies of malignant effusions. *Br. J. Cancer*, **32**, 512.

WILSON, A.P. & NEAL, F.E. (1981). *In vitro* sensitivity of human ovarian tumours to chemotherapeutic agents. *Br. J. Cancer*, **44**, 189.

WILSON, A.P., FORD, C.H.J., NEWMAN, C.E. & HOWELL, A. (1984). A comparison of three assays used for the *in vitro* chemosensitivity testing of human tumours. *Br. J. Cancer*, **49**, 57.

WILTSHAW, E. & KRONER, T. (1976). Phase II study of *cis*-dichlorodiammine platinum (II) (NSC-119875) in advanced adenocarcinoma of the ovary. *Cancer Treat. Rep.*, **60**, 55.